# Discovery of Selective Inhibitors Against EBNA1 via High Throughput *In Silico* Virtual Screening

**DOI:** 10.1371/journal.pone.0010126

**Published:** 2010-04-12

**Authors:** Ning Li, Scott Thompson, David C. Schultz, Weiliang Zhu, Hualiang Jiang, Cheng Luo, Paul M. Lieberman

**Affiliations:** 1 Drug Discovery and Design Center, State Key Laboratory of Drug Research, Shanghai Institute of Materia Medica, Chinese Academy of Sciences, Shanghai, China; 2 The Wistar Institute, Philadelphia, Pennsylvania, United States of America; Hannover Medical School, Germany

## Abstract

**Background:**

Epstein-Barr Virus (EBV) latent infection is associated with several human malignancies and is a causal agent of lymphoproliferative diseases during immunosuppression. While inhibitors of herpesvirus DNA polymerases, like gancyclovir, reduce EBV lytic cycle infection, these treatments have limited efficacy for treating latent infection. EBNA1 is an EBV-encoded DNA-binding protein required for viral genome maintenance during latent infection.

**Methodology:**

Here, we report the identification of a new class of small molecules that inhibit EBNA1 DNA binding activity. These compounds were identified by virtual screening of 90,000 low molecular mass compounds using computational docking programs with the solved crystal structure of EBNA1. Four structurally related compounds were found to inhibit EBNA1-DNA binding in biochemical assays with purified EBNA1 protein. Compounds had a range of 20–100 µM inhibition of EBNA1 in fluorescence polarization assays and were further validated for inhibition using electrophoresis mobility shift assays. These compounds exhibited no significant inhibition of an unrelated DNA binding protein. Three of these compounds inhibited EBNA1 transcription activation function in cell-based assays and reduced EBV genome copy number when incubated with a Burkitt lymphoma cell line.

**Conclusions:**

These experiments provide a proof-of-principle that virtual screening can be used to identify specific inhibitors of EBNA1 that may have potential for treatment of EBV latent infection.

## Introduction

Epstein-Barr virus (EBV) is a carcinogenic cofactor for several lymphoid and epithelial cell malignancies (reviewed in [1], [2], [3]). EBV is associated with the majority of endemic forms of Burkitt's lymphoma and nasopharyngeal carcinomas (NPC). EBV is also found in ∼40% of all Hodgkin's disease tumor biopsies, some forms of gastric carcinoma, thyroid tumors, NK/T cell lymphoma, and the majority of immunosuppression-associated non-Hodgkin's lymphomas and lymphoproliferative disease. Most EBV associated tumors harbor the latent viral genome as a multicopy episome in the nucleus of the transformed cells. During latent infection, EBV does not produce progeny virions, but does express a limited set of viral gene products that promote host-cell survival and proliferation. In proliferating cells, the maintenance of the latent viral genome depends on the functions of the Epstein-Barr Nuclear Antigen 1 (EBNA1) protein [4]. EBNA1 is expressed in all types of EBV latent infection found in proliferating cells and tumors. EBNA1 is essential for the immortalization of primary B-lymphocytes by EBV infection [5], and its inhibition by siRNA depletion or by ectopic expression of dominant negative mutants induce apoptosis in EBV-infected cells [6], [7].

EBNA1 is an attractive candidate for targeting inhibition of EBV latent infection. EBNA1 is consistently expressed in most, if not all, EBV associated malignancies[8]. EBNA1 is essential for viral genome maintenance and for infected-cell survival [6], [7]. Most importantly, EBNA1 is a viral-encoded protein that has well-defined biochemical and structural properties. EBNA1 consists of two major functional domains, a carboxy-terminal DNA binding domain, and an amino-terminal chromosome tethering domain [4], [9]. The DNA binding domain is essential for interaction with the viral origin of plasmid replication (OriP) [10]. OriP consists of a series of 30 bp repeats to which EBNA1 binds an 18 bp palindromic-sequence as a homodimer [11], [12]. The DNA binding and dimerization interface have been solved by high resolution X-ray crystallography in the apo- and DNA-bound forms [13], [14]. While there are no known cellular homologues of EBNA1, the three dimensional structure of EBNA1 resembles the overall structure of human papillomavirus (HPV) E2 protein, which has an analogous function to EBNA1 at the HPV origin of DNA replication [13]. Protein structure prediction programs suggest that EBNA1 and E2 share structural folds similar to the Kaposi's Sarcoma-Associated Herpesvirus (KSHV) LANA protein, which shares many functional properties with EBNA1, including DNA binding and episome maintenance of KSHV oriP [15]. These observations suggest that EBNA1 is a member of a family of viral origin binding proteins that have no apparent orthologue in the human genome, and therefore may represent attractive targets for inhibitors of viral latent replication and persistence.

Identification of small molecules that specifically inhibit protein-DNA binding activity has had some success [16], [17], [18], [19]. Because of the cost-inefficient and time-consuming process of conventional drug discovery over the past decade, high throughput virtual screening (HTVS) has emerged as an attractive and complementary approach to traditional solution based HTS. HTVS typically depends on the availability of a high-resolution crystal structure of the protein target as a template for computational screening. Over the years, HTVS has been applied to the successful identifications of biologically active molecules against targets such as HIV-1 protease, thymidylate, influenza hemagglutinin, and parasitic proteases [20], [21]. The availability of crystal structure of the EBNA1/DNA complex[22] presents to us an opportunity to utilize the HTVS strategy. As a proof-of-principle, we screened about 90,000 low-molecular-weight compounds from a publicly available small molecule database using the HTVS approach, and after two generations of optimization from a primary inhibitor lead, we developed a novel series of compounds with IC_50_ values in twenty micro-molar range against EBNA1. These results established our virtual screening protocol as an effective screening strategy for the discovery of potent and selective inhibitor of EBNA1, and provided a novel scaffold for future design of more potent and specific EBNA1 inhibitors.

## Results and Discussion

### High throughout virtual screening procedure

**Figure 1:**
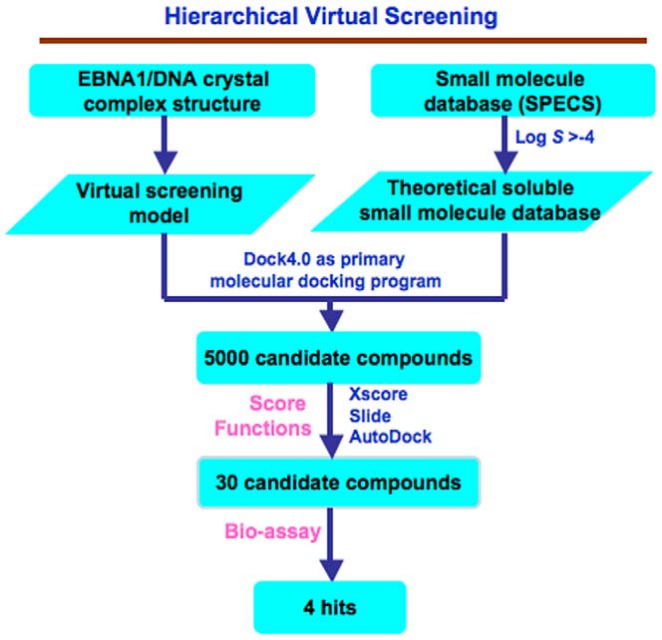
Flow chart of virtual and experimental screening strategy for discovering EBNA1 inhibitors. The EBNA1/DNA crystal structure was computationally fitted into a 6 Å grid containing every residue of the EBNA1 DNA-binding pocket was used to dock a library of compounds from the SPECS database. Compounds were preselected for solubility in an aqueous solution using a log *S* value of greater than −4. A database of ∼90,000 small-molecule compounds were then analyzed by one primary docking programs and three score functions to calculate the free energy of binding. 5000 candidates were then re-examined using Xscore, Slide, and AutoDock programs to select 30 top candidates. The top 30 compounds from 15 manually classified groups were selected for experimental DNA binding and cell-based bioassays.

**Figure 2:**
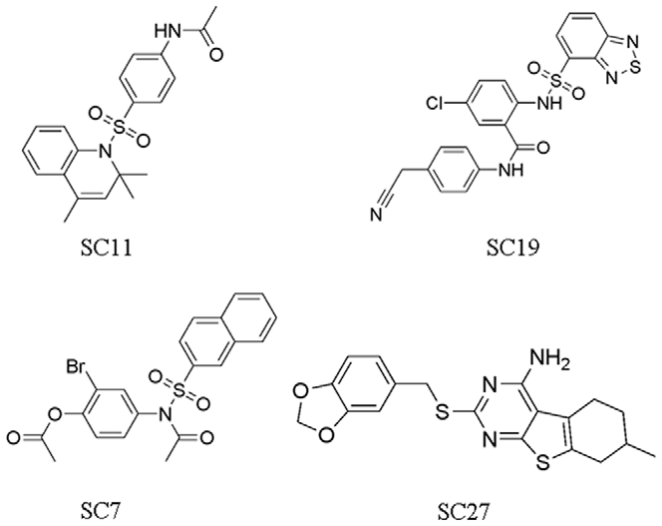
The chemical structure of four related compounds identified by virtual screening that were validated for physical inhibition of EBNA1-DNA binding.

The procedure for HTVS in this study is shown in Fig. 1. Firstly, residues within a distance of 6 Å around the DNA sequence (TGCTT) among the DNA of Epstein-Barr virus origin binding protein/DNA complexes (EBNA1, PDB entry: 1B3T) [23] were isolated for the construction of a grid for screening by the use of the DOCK4.0 program [24]. This grid was large enough to include every residue of the EBNA1 DNA-binding pocket. Next, to create a library of compounds for screening, we selected a public database that contained a large number of small molecule compounds that would be available for subsequent solution screening at a nominal cost. To this end, we selected the SPECS database that contained about 300,000 small-molecule compounds. To refine the database further to include the compounds that were likely to be soluble in an aqueous solution and enable eventual testing in solution based assays, we filtered the database for compounds with a log *S* value of greater than −4 by in-house software ZLogS, which resulted in a database of around 90,000 small-molecule compounds. Then to screen these compounds efficiently within a reasonable time, we initially used DOCK4.0 [24], a docking program that had already been successfully used for the identification of small molecule inhibitors of the HIV-1 protease, thymidylate synthase, influenza hemagglutinin, and parasitic proteases [25], as the primary molecular docking program to screen the small molecule database. The top 5000 hits that were generated from the energy scoring function of DOCK4.0 were docked using three other docking programs that employed different scoring functions. XScore [26] (version 1.2.1) was used for calculating a binding score for a given protein-ligand complex structure, SLIDE (version 2.3.1) [27], [28] was used for the calculation of hydrogen bonds and the hydrophobic complementarity while considering the flexibility of both protein and ligand, and AutoDock3.0[29] was used for calculating the free energy of binding. Specifically, the XScore program was first carried out on the top 5000 candidate compounds that were generated from DOCK4.0. The top 2000 compounds from XScore were then selected for reevaluation by the use of the SLIDE scoring function. The top 500 potential hits from SLIDE were finally evaluated according to the free energy of binding with the AutoDock3.0 program. According to their binding modes, free-energy scores, and scaffold diversity, finally, 30 compounds from 15 manually classified groups were selected for experimental validation. The 30 compounds were assayed by fluorescence polarization (FP) and electrophoresis mobility shift assay (EMSA) for physical inhibition of EBNA1-DNA binding (data not shown). As a control for specificity, the compounds were rescreened for there inhibition of an unrelated DNA binding protein, Zta, also encoded by EBV. Zta is an EBV-encoded b-zip DNA binding protein that bears no structural resemblance to EBNA1 and is unlikely to be affected by EBNA1-specific inhibitors. Among the 30 candidates, four compounds were found to have selective inhibitory activity for EBNA1 and not for Zta. The structure of the four compounds, referred to as SC7, SC11, SC19, and SC27 are shown in Fig. 2.

### Biochemical validation of EBNA1 Inhibitors Identified through Virtual Screening

**Figure 3:**
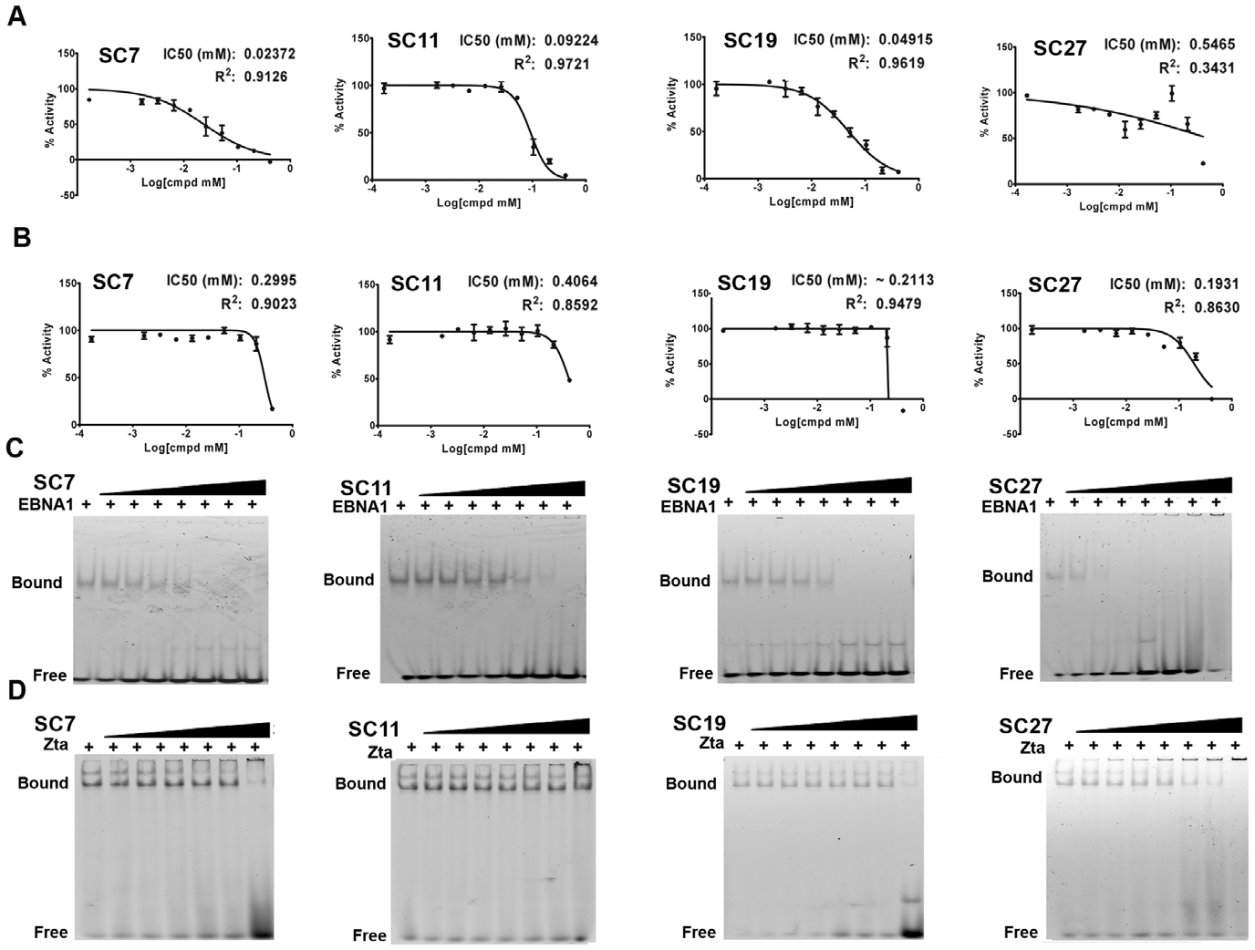
Physical inhibition of EBNA1-DNA binding assays. Candidate inhibitors SC7, SC11, SC19, and SC27 were assayed by fluorescence polarization (FP) for inhibition of EBNA1-DNA binding (panel A) and for inhibition of Zta-DNA binding (panel B). IC50 values were calculated for each isotherm. Inhibitor concentrations were diluted 2-fold from 833 to 7 µM for each compound. Inhibitors were also assayed using a secondary EMSA assay to monitor EBNA1-DNA binding (panel C) or Zta-DNA binding (panel D) using the same concentrations of inhibitor compounds (two fold dilutions from 833 to 7 µM) as that shown for FP assays in panels A and B, above.

The four candidate compounds were further characterized for their relative potency of inhibiting EBNA1-DNA binding using both the FP and EMSA. EBNA1 DNA binding domain was purified to near homogeneity and capable of binding a fluorescent tagged DNA hairpin containing a consensus EBNA1 binding site with ∼50 nM affinity (data not shown). As a control for selectivity, the same analysis was performed with purified Zta, and its cognate fluorescent DNA probe. In the FP assay, SC7, SC11, and SC19 had IC_50_ ranges between 20–100 µM, while SC27 did not perform well in the FP assay. However, SC27 showed highly specific inhibition of EBNA1 in EMSA (Fig. 3D, lower right panel), suggesting that the poor performance in FP may be due to fluorescence interference properties or solution solubility problems. The EMSA analysis confirmed that each compound inhibited EBNA1-DNA binding with an IC50 between 20–100 µM, similar to that determined by FP. SC7, SC11, and SC19 showed no significant inhibition of Zta-DNA binding in either the FP assay or EMSA, suggesting that these compounds are selective inhibitors of EBNA1-DNA binding function.

### Inhibition of EBNA1 functions in cell-based assays

**Figure 4:**
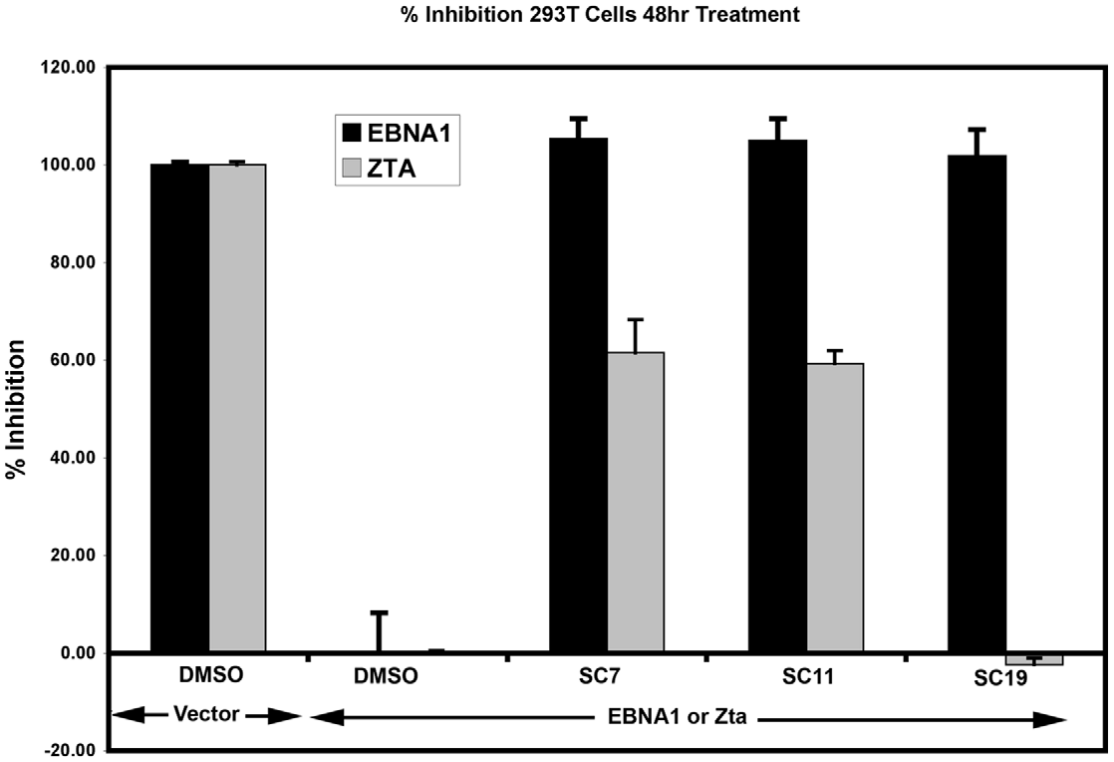
Inhibition of EBNA1 transcription activation function in cell-based assays. Soluble compounds SC7, SC11, and SC19 were assayed for their ability to inhibit either EBNA1 or Zta-dependent transcription activation in transfected 293T cells. Cells were transfected with vector or EBNA1 expression plasmid and the OriP-Cp-Luciferase reporter plasmid in the presence of 5 µM SC7, SC11, SC19 or DMSO control. 100% inhibition was equivalent to basal expression levels of OriP-Cp-Luc in the absence of ectopic EBNA1 expression. In parallel experiments, the same compounds were also assayed for inhibition of Zta transcription activation of the BHLF1-Luciferase reporter plasmid. Percent inhibition of Zta is shown in grey. Percent inhibition of EBNA1 is shown in black. Error bars represent standard deviation from the mean for at least three experimental replicates.

SC7, SC11, and SC19 were assayed for their ability to inhibit EBNA1 in two cell-based assays. Transcription activation of the EBV Cp promoter by EBNA1 is known to be essential for B-cell immortalization [30]. We therefore assayed the effect of compounds to inhibit EBNA1 transcription activation of Cp using a luciferase reporter-based assay in transiently transfected HEK293T cells (Fig. 4). The luciferase reporter plasmid consisted of a ∼2 kb region of EBV containing both OriP and Cp fused upstream of the luciferase gene. Cotransfection of this plasmid with an EBNA1 expression plasmid produced a 4–5 fold increase in luciferase activity relative to control expression vector lacking EBNA1 (data not shown). Incubation of transfected cells with control vehicle DMSO had no effect on EBNA1 transcription activation. Incubation of transfected cells with 5 µM SC7, SC11, or SC19 completely blocked EBNA1 transcription activation (Fig. 4). As a control for specificity, the same compounds were assayed for their inhibition of Zta transcription activation. Zta is a potent transcription activator of the EBV BHLF1 promoter. We therefore assayed these compounds for their effect on Zta transactivation of BHLF1 promoter fused to luciferase reporter gene. Cells incubated with 5 µM SC7 and S11 showed ∼60% inhibition of Zta transactivation, indicating that these compounds were not highly selective inhibitors of EBNA1 transcription function in vivo. In contrast, cells incubated with 5 µM SC19 had no detectable inhibitory effect on Zta transactivation, yet a robust inhibition of EBNA1. This indicates that SC19 can selectively inhibit EBNA1 transcription activation function in a cell-based assay.

### Elimination of EBV episomes

**Figure 5:**
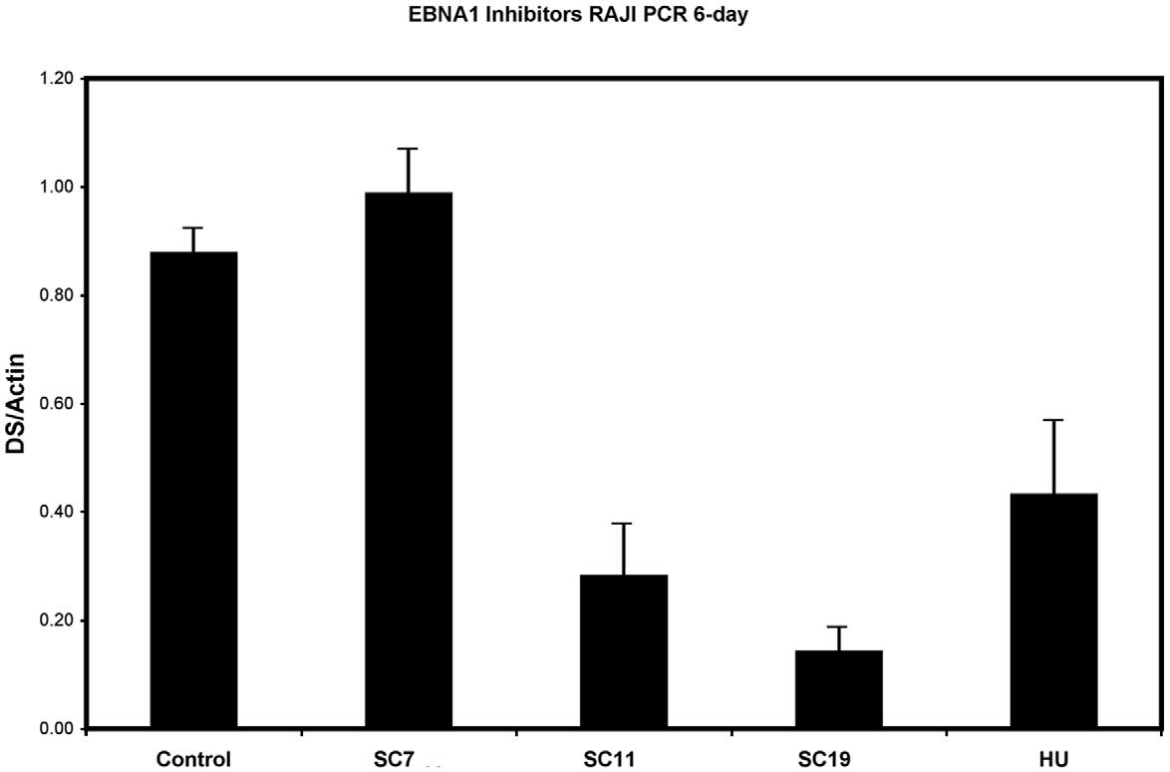
Elimination of EBV genomes from Burkitt lymphoma cell lines. EBV-positive Raji Burkitt lymphoma cell lines were treated with 10 µM SC7, SC11, or SC19 or DMSO control for six days. EBV genome copy number was determined by quantitative real-time PCR analysis of EBV DNA (DS) relative to cellular DNA (actin). Error bars represent standard deviation from the mean for at least three experimental replicates.

To further evaluate the ability of these compounds to inhibit the EBNA1 function required for EBV genome replication, we assayed their effect on EBV genome copy number in the Raji Burkitt lymphoma cell line (Fig. 5). Raji cells typically contain ∼100 copies of the EBV genome per cell. Previous studies have demonstrated that hydroxyurea (HU) can reduce the number of EBV episomes in Burkitt lymphoma cell lines, including Raji cells. Therefore, we assessed the effects of HU, SC7, SC11, and SC19 on EBV copy number in Raji cells. Cells were treated with 10 µM SC7, SC11, SC19 or with 100 µM HU for six days. The EBV genome copy number was determined by real time PCR for EBV DNA (DS) relative to cellular actin DNA. We found that HU treatment caused ∼50% reduction in EBV genome copy number. Treatment with SC11 or SC19 caused a 75–90% reduction in EBV copy number, while SC7 had no apparent effect (Fig. 5). These findings suggest that SC11 and SC19 might be more effective than HU in promoting loss of EBV genomes from latently infected cells.

### Molecular docking interaction analysis

**Figure 6:**
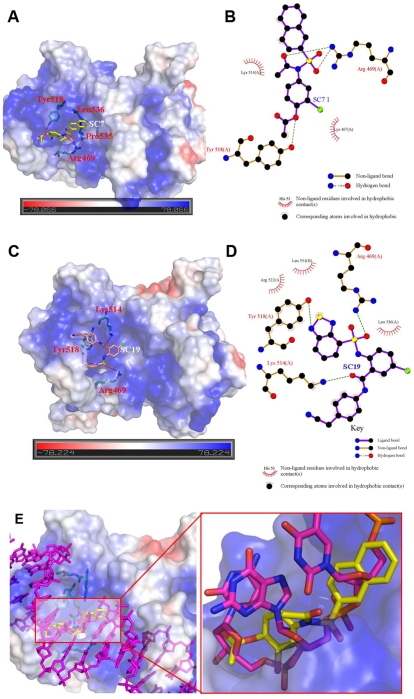
Docking simulation of two best hits (SC7 and SC19) in the EBNA1 site. (A) Interactions of SC7 with the EBNA1 binding Pocket; (B) Interaction analysis between SC7 and EBNA1 calculated by LIGPLOT program; (C) Interactions of SC19 with the EBNA1 binding Pocket; (D) Interaction analysis between SC19 and EBNA1 calculated by LIGPLOT program; (E) The comparison between the binding mode of SC7/EBNA1 and DNA/EBNA1 crystal complex structure, SC7 is shown in yellow stick and DNA is shown in magenta stick.

**Figure 7:**
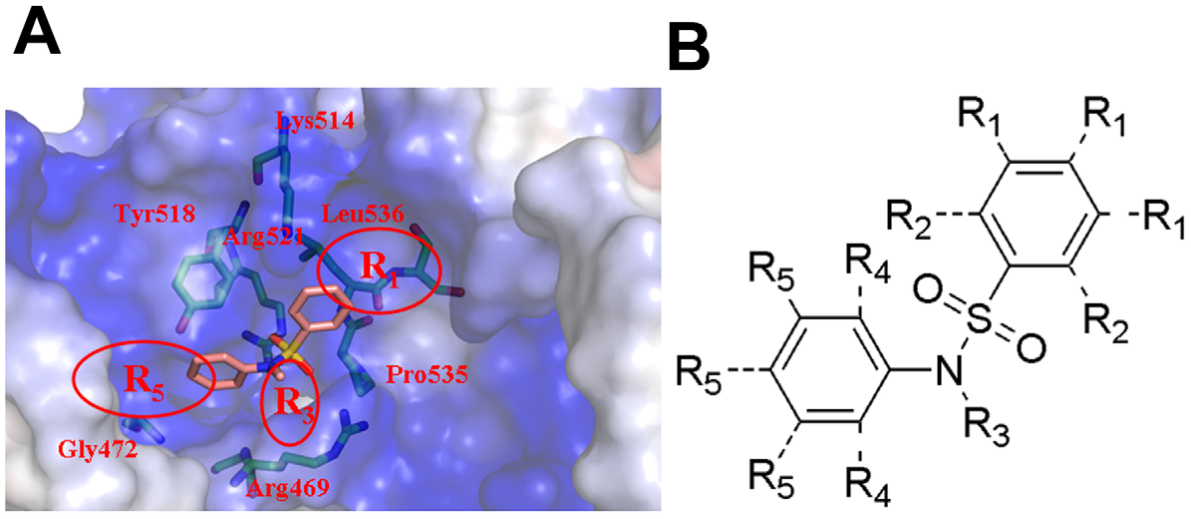
Design strategy for EBNA1 inhibitors with greater potency and specificity. (A) Corresponding position of three modification sites within the DNA binding pocket of EBNA1; (B) Base scaffold with three possible positions for modification.

To understand the mode of inhibition of compounds binding with DNA binding site of EBNA1, we docked the best two inhibitors (SC7 and SC19, their inhibitory activity against EBNA1 is 23 and 49 µM, respectively) into the DNA binding site of EBNA1 by using AutoDock3.0, shown in Fig. 6. As shown in Fig. 6A, SC7 is modeled to bind the DNA-binding site of EBNA1 that forms two hydrogen bonds with the nitrogen of side chain of Arg469 and one hydrogen bond with oxygen of side-chain of Tyr518, respectively. In addition, the binding mode suggests that extensive hydrophobic interactions are formed between SC7 and hydrophobic region (R1 region in Fig. 7) near the residues Pro535 and Leu536. In particular, from the electrostatic surface contour analysis shown in Fig. 6A, SC7 matches the binding site very well inwhich the hydrophobic motif of SC7 interacts with hydrophobic regions of EBNA1, while the negative-charged oxygen of sulfonyl and bromide in 2-bromophenyl acetate motifs interact with the positive charged region near the Arg469 (R3 region in Fig. 7). In comparison with the EBNA1-DNA crystal structure, the SC7 is well aligned with the T111, G112 and C113 of the DNA sequence (shown in Fig. 6E). In particular, the sulfonyl motif mimics the interaction with the receptor as the phosphate motif of G112 of DNA sequence. In contrast to SC7, SC19 is modeled to bind to the EBNA1 active site in a different orientation, which is apparently due to the bulky phenyl group derivatization on the benzamide motif (SC19) (Fig. 6C and D). In addition to extensive hydrophobic interactions between EBNA1 and SC19 with residues Lys514, Tyr518, Arg522, Leu536 and Leu554, SC19 also makes three additional hydrogen bonding interactions with Arg469, Lys514 and Tyr518 of EBNA1. Based on the interaction analysis of SC7 and SC19 binding with EBNA1, the residue Arg469 and Tyr518 may play crucial role in the maintaining the inhibitory activity of inhibitor binding with EBNA1.

### Conclusions and Future Prospects

We have identified four small molecules as novel inhibitors against EBNA1 by using a HTVS approach from a filtered small molecule SPECS compound database that contains about 90,000 compounds. Among these novel inhibitors, compound **SC7** was found to inhibit the DNA binding activity of the EBNA1 protein in FP assays with an IC_50_ value of 23 µM. On the basis of the molecular docking of this compound to EBNA1, a fragment scaffold (the scaffold listed in Fig. 6B) was hypothesized to be the functional moiety for EBNA1 inhibition. Furthermore, the docking simulation of SC7 and SC19 into the EBNA1 active site provides some pharmacophore clues for future inhibitor optimization to increase EBNA1 inhibitor potency and selectivity (Fig. 6 or 7). In particular, compounds with modifications that extend the R1 position of the phenyl group with hydrophobic motifs might enable compound analogues to reach deeper into the EBNA1-binding pocket that interacts with residue Pro535 and Leu536 of EBNA1. The modification of bulky group at the R2 and R4 positions may clash with the amino acid side-chain residues of the EBNA1 DNA binding pocket, therefore changes at these positions may be limited to relatively small substituents. In the R3 position, the substitution of a bulky group, such as a phenyl, will likely be unfavorable for the binding of the inhibitor to EBNA1. In contrast, hydrophilic groups introduced at the R5 and R3 groups could mediate additional hydrogen bonding interactions with the residues of EBNA1 protein (Arg469 in R3 position, Gly472 and Tyr518 in R5 position) and potential enhance the binding affinity of the inhibitors.

Our study demonstrates that an efficient and cost-effective virtual screening procedure can be used to identify novel EBNA1 inhibitors that also show considerable selectivity for EBNA1 over other proteins. Moreover, the promising results that were obtained in this study will serve as an excellent platform for the further development of EBNA1 inhibitors with even greater potency and selectivity for use as therapeutic agents against latent EBV infection.

## Materials and Methods

### Virtual screening

The virtual screening strategy was shown in Fig. 1. The X-ray crystal structure of the Epstein-Barr virus origin binding protein/DNA complexes (EBNA1, PDB accession code 1B3T) [23] was used as the target structure in this approach. The modified small molecular database containing approximately 90,000 molecules for virtual screening was generated as a SPECS subset from the Zinc databases (compounds are available from the SPECS Company)[31] with a predicted solubility filter by in-house program ZLogS (log *S*>−4). ZLogS (unpublished) was gifted from Dr. MY Zheng in Drug Discovery and Design Center, SIMM, Chinese Academy of Sciences. ZlogS performs solubility prediction based on the generalized atom additive model and stepwise multiple linear regression (SMLR). Eight putative relationships between the atomic solvent assessable surface area, electro-descriptors and atomic contribution of water solubility were investigated.

The virtual screening was performed on SGI Origin3800 computer at the Shanghai Institute of Materia Medica. A heuristic docking and consensus scoring strategy was used to evaluate the results of the virtual screening. Specifically, we used DOCK4.0 as the primary screening tool targeting at DNA binding site of the X-ray crystal structure of EBNA1. Residues around the DNA at radius of 6 Å was isolated for the construction of the grid for docking simulation. This radius was large enough to include all of the residues that are involved in putative inhibitor site. During the docking procedure, Kollman-all-atom charges were assigned to the protein, and Geisterger-Hückel charges assigned to the small molecules in the SPECS database. Furthermore, the conformational flexibility of the molecules from the database was considered during the docking simulations. We used 30 configurations per ligand building a cycle and 50 maximum anchor orientations were used in the anchor-first docking algorithm. After the protocol was set up, the modified database was screened and top-5000 molecules were taken as the hits list for further analyses. These molecules were re-ranked by Xscore (version 1.2.1)[26] and top-2000 molecules were taken as the hits list for the docking and scoring mode of SLIDE (version 2.3.1)[27], [28]. The binding affinity of identified top-500 potential hits from SLIDE were further evaluated by AutoDock3.0[29]. Last, on the basis of the results of these scoring functions, the top 200 molecules were extracted and carefully considered for the receptor binding and scaffold diversity. Finally, we purchased 30 available candidate compounds from different scaffolds for the in vitro assay.

### Score functions with Xscore, SLIDE and Autodock

In this study, the Xscore and SLIDE were performed as score functions with default parameters. And last, the molecular docking program AutoDock 3.0[29] was used for the automated molecular docking simulations for the prediction of the binding affinity. The docking scheme is summarized as follows. First, the receptor molecule was checked for polar hydrogen and was assigned for partial atomic charges. The PDBQS file was created, and the atomic solvation parameters were also assigned. Second, a 3D search grid was created by the use of AutoGrid algorithm[32] to evaluate the binding energies between the ligands and the EBNA1 receptors. Third, a series of the docking parameters were defined. The atom types, generations and the run numbers for LGA algorithm were properly assigned according to the requirement of the Amber force field. The number of generations, energy evaluations, and docking runs were set to 370,000, 1,500,000, and 20, respectively. Kollman all-atom charges were assigned for the EBNA1 receptors and Gasteiger-Marsili [22] charges were assigned for the ligands. Finally, the docked ligand-receptor complexes were selected according to the criteria of interacting energy combined with geometrical matching quality. These complexes were used as the starting conformation for further energetic minimization and geometrical optimization before the final binding models were achieved.

### Compounds

All compounds used were ordered from SPECS company which reported purities of over 90% for all compounds as analyzed by LCMS. Compounds were stored as powder in a dessicator and resuspended to 50 mM in DMSO immediately prior to use. SPECS catalogue numbers are as follows: SC7: AG-690/36423053; SC11: AG-690/36749014; SC19: AG-690/36535028; SC27: AJ-292/40706570.

### Protein Purification

The EBNA1 DNA binding domain (DBD) (aa 454–607) with a hexa-histidine amino-terminal fusion protein was expressed in E. coli and purified over Ni-NTA agarose according to manufacturers recommendations (Qiagen). The protein was purified from four liters of E. coli to generate ∼20 mg of highly purified EBNA1 protein, estimated to be >90% pure. Purified protein was then dialyzed into a buffer consisting of 200 mM NaCl, 20 mM, Tris-HCl pH 7.4 and 20% Glycerol. Hexa-His tagged Zta was purified from E. coli to near homogeneity, similar to EBNA1 protein. Purified Zta was dialyzed into 500 mM NaCl, Tris-HCl pH 7.4 and 20% Glycerol.

### FP Assay EBNA1

A reaction mix containing 200 mM NaCl, 20 mM Tris-Cl pH 7.4, 1 mM DTT, 10 ug/mL BSA, 10 nM Cy5 5′ modified EBNA1 (Cy5-GGGTAGCATATGCTATCTagatagcatatgctaccc) or ZTA (Cy5-CACTGACTCATTaatgagtcagtg binding site) oligonucleotide hairpins (purchased from IDT) and 246 nM EBNA1 DBD (aa 459–607) or 300 nM ZTA full length purified recombinant protein was incubated for 20 minutes at room temperature prior to dispensing (BioTek MicroFlo Select) 30 uL to each well of a 384 well black opaque microtiter plate containing the test compounds. Test plates were centrifuged at 165×g prior to fluoresence polarizaration measurements in an Envision Xciter multilable plate reader (Perkin Elmer) using a Cy5 FP 620 excitation and Cy5 FP P/S-pol 688 emission filters.

### EMSA Assay

An EMSA reaction buffer was prepared containing 20% Glycerol, 200 mM NaCl, 20 mM Tris-Cl pH 7.4, 1 mM DTT, 10 ug/mL BSA, 10 nM Cy5 oligonucleotide probe and with or without 246 nM EBNA1 DBD or 300 nM Zta full length purified recombinant protein. This solution was incubated for 20 minutes at room temperature to ensure binding. 30 uL of this solution was dispensed to eppendorf tubes containing 0.5 uL of a test compound in DMSO and mixed. Samples were then loaded onto a 6% polyacrylamide gel in 1/2X Tris-Borate buffer and electrophoresed for 90 min at 170 V. The gel was then scanned for flouresence using at Typhoon Imager (GE Healthcare).

### FP screening

For initial screening 2 ul of test compounds resuspended in DMSO were plated in triplicate wells in a black opaque Optiplate (Perkin Elmer) at a concentration of 50 mM to achieve a final concentration of 3.33 mM when resuspended in 30 uL of reaction solution. The plate was assayed using the FP assay above.

### IC_50_ Determination

To determine IC50s, an 11- point series of 2-fold dilutions in DMSO was performed in duplicate starting at an initial concentration of 50 mM using a Janus Verispan (Perkin Elmer) to create a master plate. From this master plate 0.5 uL was transfered to black opaque 384 well Optiplate (Perkin Elmer) using a Janus MDT (Perkin Elmer) or to microfuge tubes by hand. The plate or microfuge tubes were assayed using the FP and EMSA protocols described above. IC50 plots were generated by analyzing the binding of EBNA1 or Zta with inhibitor compounds ranging from 833 to 7 uM final concentrations following two fold dilutions (specifically 833, 417, 208, 104, 52, 26, 13, 7, and 0 µM). The data was calculated as percentage of EBNA1 binding activity and then plotted versus the log of the concentration of inhibitor. Using GraphPad Prism 5.0 software the plotted data was fit with a Hill-Slope dose-response curve to calculate the relative IC50.

### Raji EBV Genome Copy Number Assay

Raji cells (purchased from ATCC) were grown at 5×10^6^ cells/ml in RPMI media supplemented with 10% FBS and 10 mM Streptomycin and 10 mm Penicillin. Test compounds were added to achieve a final concentration of 10 uM. Cells were grown at 37°C for three days and then passaged 1∶10 into fresh media with the same concentration of drug. After a second 3-day incubation cells were harvested and the DNA was isolated using ChIP Lysis Buffer followed by sonication, phenol∶chloroform extraction, and ethanol precipitation. Relative EBV DNA copy number was quantified by real-time PCR with primers for cellular actin and the EBV dyad-symmetry region, as described previously [33].

### EBNA1 Transcription Activation Assay

HEK293T cells (purchased from ATCC) were seeded in 24-well plates at a density of 50,000 cells/well in DMEM media with 10% FBS. Following an 18 hr incubation at 37°C, cells were transfected using 2 µl of lipofectamine with 0.4 ug/well of a pCMV-FLAG-EBNA1, pCMV-FLAG-ZTA (N362) or a control pCMV-FLAG vector and 0.2 ug/well of either a Cp-Luciferase (EBNA1-reporter) or a H_P_-Luciferase (Zta-reporter) plasmid. All samples were cotransfected with 100 ng of Renilla expression vector as an internal control for transfection efficiency. After 6 hrs the transfection media was replaced and test compounds were added to achieve a final concentration of 5 uM. Cells were incubated at 37°C for 48 hrs and then harvested. Cells were lysed and prepared for analysis using the Promega Dual Reporter system and luminescence was measured using a Perkin Elmer Wallac Victor^2^ 1420 Multilabel reader.
